# Draft Genome Sequences of Eight Strains of Campylobacter helveticus Isolated from Cats and a Dog in New Zealand

**DOI:** 10.1128/MRA.01244-19

**Published:** 2020-01-30

**Authors:** Krunoslav Bojanić, Anne C. Midwinter, Els Acke, Jonathan C. Marshall, Angela J. Cornelius, Patrick J. Biggs

**Affiliations:** a School of Veterinary Science, Massey University, Palmerston North, New Zealand; b School of Fundamental Sciences, Massey University, Palmerston North, New Zealand; c Institute of Environmental Science and Research Limited, Christchurch, New Zealand; d New Zealand Food Safety Science and Research Centre, Massey University, Palmerston North, New Zealand; University of Maryland School of Medicine

## Abstract

The draft genome sequences for eight isolates of Campylobacter helveticus isolated from companion animals are described and compared with that of the type strain. On average, the genomes are 1,825,025 bp long and have a GC content of 34.4% and 1,885 coding DNA sequences (CDSs). CRISPRs were detected in only one isolate and phages in none.

## ANNOUNCEMENT

Campylobacter helveticus has been isolated from healthy and diarrheic companion animals and is closely related to C. upsaliensis (1). However, *C. helveticus* has not been isolated from humans with gastroenteritis (2). In a study exploring *Campylobacter* spp. in companion animals and their food (3) (approved by the Massey University Animal Ethics Committee; application number 09/70), *C. helveticus* isolates from clinically normal cats (seven isolates, one from each) and a dog (one isolate) were sequenced and analyzed with the genome of *C. helveticus* strain ATCC 51209^T^ (4).

Bacteria were isolated and identified as described (3) with subculture onto Columbia horse blood agar (Fort Richard Laboratories, New Zealand) in an H_2_-enriched microaerobic atmosphere at 37°C. Genomic DNA was extracted from the Columbia horse blood agar subcultures using a QIAamp DNA minikit (Qiagen, Germany) and sequenced at New Zealand Genomics Ltd. (Massey University, New Zealand) using a MiSeq instrument (Illumina, Australia) following the manufacturer’s instructions, with paired read lengths of 250 base pairs after library preparation using the Nextera XT library kit (Illumina).

Default parameters and settings were used for all software unless otherwise described. Velvet version 1.2.10 (5) was used for *de novo* genome assembly. Sequence data were quality processed, analyzed, and assembled using ea-utils (https://bio.tools/ea-utils) within QCtool (https://bio.tools/qctool) and SPAdes version 3.13.0 (6). Assembly statistics for the eight New Zealand isolates were extracted from the NCBI Prokaryotic Genome Annotation Pipeline (PGAP) and are described in Table 1. Geneious version 10.2.6 (7) was used to calculate GC values and to check for the presence of the *cdtABC* operon. Signal peptides from amino acid predictions were discovered using the SignalP-5.0 server (http://www.cbs.dtu.dk/services/SignalP-5.0/).

**TABLE 1:** Assembly information for eight Campylobacter helveticus isolates from New Zealand

Parameter	Data for strain:							
	ACP102b	ACP108a	ACP110b	ACP114b	ACP123b	ACP141a	ACP175a	ACP183a
No. of reads	2,619,932	2,218,260	1,991,084	1,821,760	1,155,192	1,211,896	1,386,372	2,157,198
No. of contigs	149	185	180	175	181	155	149	161
Genome length (bp)	1,768,011	1,881,742	1,859,862	1,784,884	1,899,321	1,821,144	1,829,303	1,755,938
*N*_50_ (bp)	29,027	30,102	26,651	33,943	28,602	30,920	32,673	30,818
Coverage (×)	360	309	277	247	159	168	192	296
GC content (%)	34.46	34.27	34.31	34.34	34.22	34.4	34.39	34.53
No. of proteins	1,791	1,950	1,941	1,830	1,986	1,882	1,888	1,814
No. of rRNAs	3	5	6	5	4	11	9	6
No. of tRNAs	39	34	34	39	36	35	33	39
No. of other RNAs	3	3	3	3	3	3	3	3
No. of genes	1,897	2,058	2,043	1,935	2,097	2,000	2,006	1,923
No. of signal peptides	126	146	148	135	152	139	137	137
GenBank accession no.	VDBW00000000	VDBV00000000	VDBU00000000	VDBT00000000	VDBS00000000	VDBR00000000	VDBQ00000000	VDBP00000000
SRA run no.	SRR10248249	SRR10248248	SRR10248247	SRR10248246	SRR10248244	SRR10248243	SRR10248242	SRR10248245
BioSample no.	SAMN11567273	SAMN11567274	SAMN11567275	SAMN11567276	SAMN11567277	SAMN11567278	SAMN11567279	SAMN11567280
SRA no.	SRX6966295	SRX6966296	SRX6966297	SRX6966298	SRX6966299	SRX6966300	SRX6966301	SRX6966302
Host	Felis catus	Felis catus	Felis catus	Felis catus	Felis catus	Canis lupus familiarus	Felis catus	Felis catus

For functional and core genome analysis, concatenated contigs were annotated with Prokka version 1.11 (8) using the default settings and the predicted amino acid sequences were searched against the Clusters of Orthologous Groups (COGs) database (using COGsoft version 201204) (9). COGs were considered to be core if present in all of the isolates. CRISPRFinder (10) and PHAST (11) were used to identify clustered regularly interspaced short palindromic repeats (CRISPRs) and phage, respectively. Abricate version 0.8.11 (https://github.com/tseemann/abricate) was used via Nullarbor to screen for genes related to antimicrobial resistance.

Aligning the coding DNA sequences (CDSs) to the COGs database gave 1,243 unique COGs in the pangenome of the eight isolates plus the reference genome, with an average 1,169 COGs and 1.34 copies per genome. The *C. helveticus* core genome contained 1,083 COGs, approximately 87.1% of the pangenome. CRISPRs were detected in one isolate (ACP114b), although the other isolates had 1 to 3 “questionable” CRISPRs detected. No intact bacteriophage insertions were identified. Only two genes associated with antimicrobial resistance were found, both in ACP110b. The *cdtABC* operon (cytolethal distending toxin) was detected in all eight isolates.

### Data availability.

The *C. helveticus* draft genome sequences have been deposited in GenBank under the BioProject accession number PRJNA541328, with the individual accession numbers shown in Table 1.
